# Working together: reflections on how to make public involvement in research work

**DOI:** 10.1186/s40900-023-00427-4

**Published:** 2023-03-25

**Authors:** Lynn McVey, Tina Frost, Basma Issa, Eva Davison, Jamil Abdulkader, Rebecca Randell, Natasha Alvarado, Hadar Zaman, Nicholas Hardiker, V.-Lin Cheong, David Woodcock

**Affiliations:** 1 Faculty of Health Studies, University of Bradford, Bradford, UK; 2 Wolfson Centre for Applied Health Research, Bradford, UK; 3 University of Huddersfield, Huddersfield, UK; 4 Pharmacist Elderly and Interfaces of Care, Medicines Management and Pharmacy Services, Leeds Teaching Hospitals Trust and School of Healthcare, University of Leeds, Leeds, UK

**Keywords:** Experiential expertise, Patient and public involvement, Reflective accounts, Service user researcher, Coproduction

## Abstract

**Background:**

The importance of involving members of the public in the development, implementation and dissemination of research is increasingly recognised. There have been calls to share examples of how this can be done, and this paper responds by reporting how professional and lay researchers collaborated on a research study about falls prevention among older patients in English acute hospitals. It focuses on how they worked together in ways that valued all contributions, as envisaged in the UK standards for public involvement for better health and social care research.

**Methods:**

The paper is itself an example of working together, having been written by a team of lay and professional researchers. It draws on empirical evidence from evaluations they carried out about the extent to which the study took patient and public perspectives into account, as well as reflective statements they produced as co-authors, which, in turn, contributed to the end-of-project evaluation.

**Results:**

Lay contributors’ deep involvement in the research had a positive effect on the project and the individuals involved, but there were also difficulties. Positive impacts included lay contributors focusing the project on areas that matter most to patients and their families, improving the quality and relevance of outcomes by contributing to data analysis, and feeling they were ‘honouring’ their personal experience of the subject of study. Negative impacts included the potential for lay people to feel overwhelmed by the challenges involved in achieving the societal or organisational changes necessary to address research issues, which can cause them to question their rationale for public involvement.

**Conclusions:**

The paper concludes with practical recommendations for working together effectively in research. These cover the need to discuss the potential emotional impacts of such work with lay candidates during recruitment and induction and to support lay people with these impacts throughout projects; finding ways to address power imbalances and practical challenges; and tips on facilitating processes within lay groups, especially relational processes like the development of mutual trust.

## Abstract (summary)

Involving members of the public in all stages of research as equal partners is a powerful way to make research more relevant. This paper shares an example of such involvement, from a study about falls prevention in English hospitals. Developed by a team of lay people and professional researchers, the paper looks at how we worked together, drawing on evaluations we carried out about how the study took patient and public perspectives into account, and on personal reflections we wrote. Public involvement had a positive effect on the project and the individuals involved, but there were also difficulties. Positive impacts included lay people ensuring the study focused on what mattered most to patients and their families and feeling they had done right by their personal experience of the study’s subject. Negative impacts included the potential for people to feel overwhelmed by the changes in organisations or in wider society needed to address the issues being explored by a research study, which could cause them to question why they became involved in the first place. The paper ends with practical recommendations about working together, covering things such as helping lay people with the emotional impact of involvement from the beginning to the end of projects; finding ways to ensure everybody is treated in the same way and solving practical problems; and tips on leading and supporting groups of this kind, especially with personal issues like trusting each other.

## Background

The importance of involving members of the public in the development, implementation and dissemination of research is recognised increasingly world-wide, particularly in the field of health [1, 2]. The UK is regarded as a leader in the field [3, 4]; the National Institute for Health and Care Research (NIHR) in England, for example, requires applicants to explain how public contributors will be involved in all stages of the studies it funds, from design and operation to evaluating impact [3, 5]. Guidance and standards have been developed to support such work [6–8], with a growing focus on partnership approaches, such as coproduction [3, 6], a method ‘*in which researchers, practitioners and the public work together, sharing power and responsibility from the start to the end of the project*’ [6] (p.2). Indeed, working together in ways that value all contributions, and that build and sustain mutually respectful and productive relationships is one of the six UK standards for public involvement for better health and social care research [8].

Partnerships of this kind involve people working together sensitively and respectfully and are therefore a relational undertaking, underpinned by the development of trust between members of the public and researchers [6]. Such relationships require dialogue, empathy, reciprocity and openness [6, 9]: positive interpersonal qualities that have led to approaches like coproduction being called a ‘kind revolution’ [10]. But trust does not grow simply from academics being ‘kind’ to lay colleagues, a notion that hints at the power imbalances that can be inherent in such relationships. It also requires an acknowledgement of these imbalances and the tensions they can create, and the development of new, less hierarchical structures in which power is shared [1, 2, 6, 9, 11]. Given prevailing financial, political, organisational and cultural forces, several authors have written about the difficulties of achieving such relational ends [1, 4, 11–13]. Powerful emotions can be aroused, both for public contributors sharing learning from what can be painful lived experiences [13], and for professional researchers working with them, who, Boylan et al. [11] found, undertake emotional labour too, whether when seeking to care for their lay fellows or when dealing with their own feelings, which they may try to suppress.

Nevertheless, the claimed benefits of such partnerships are considerable, ranging from improving the quality and relevance of research to achieving democratic, ethical ways of working as moral ends in themselves [1, 4, 5, 12]. As an example, in a large randomised control trial known as the 3D study, which aimed to improve the care of people with multiple long-term conditions, a patient involvement group contributed strongly from the beginning to the end of the research, for instance by helping to develop a new measure of treatment burden [14]. Such cases notwithstanding, more could be done to evidence impacts [12] and calls to share ‘*models and examples of flexible practice that ensure a strong patient voice at all stages of research*’ [3] (p.28) have been made. Given the relational basis of such work, a particular need has been identified for examples that attend to the interpersonal dynamics of public involvement [2]. This paper responds to such calls, by reporting how professional researchers and public contributors (henceforth referred to as *lay researchers*, as they are in the project described here)^1^ have worked together on a NIHR-funded study about falls prevention among older patients in English acute hospitals [15]. It focuses on our progress towards achieving effective, patient- and person-centred public involvement, as envisaged in the UK standards [8], particularly the standard ‘working together’, and on what we have learned as we have done so. We pay particular attention to the relational processes that contributed to this—what it felt like for us to be partners in this approach—and how knowledge generated by the project has been impacted by our relationships. We believe this emphasis on the nature of the interactions between lay and professional researchers, sustained from the beginning to the end of the project, enables it to make a distinctive contribution to the literature.

## Methods

This paper is itself a product of *working together*, having been produced by a team of lay (DW, TF, BI, ED and JA) and professional researchers (RR, LM, NA, HZ, NH and VC), drawing on empirical evidence from evaluations carried out as part of the project, and on reflective statements some of us wrote as co-authors, which, in turn, contributed to the end-of-project evaluation. Below, we provide contextual information about the research project and how lay involvement was integrated within it, and about the evaluative and reflective methods used.

### The FRAMES project

This NIHR-funded research project was an investigation of falls risk assessment and prevention among older patients in acute hospitals in England, described in more detail elsewhere [15]. It was given the acronym *FRAMES* (**F**alls prevention in older adults: using **R**ealist **A**pproaches to improve **M**ultifactorial ass**E**ssment and intervention**S**), a name chosen by lay researchers from a shortlist produced by the study’s project management group (comprising researchers and co-investigators, including the lay lead, DW). The acronym was, in fact, one devised by the lay lead (DW), although the other lay researchers did not know that when they selected it.

**Fig. 1:**
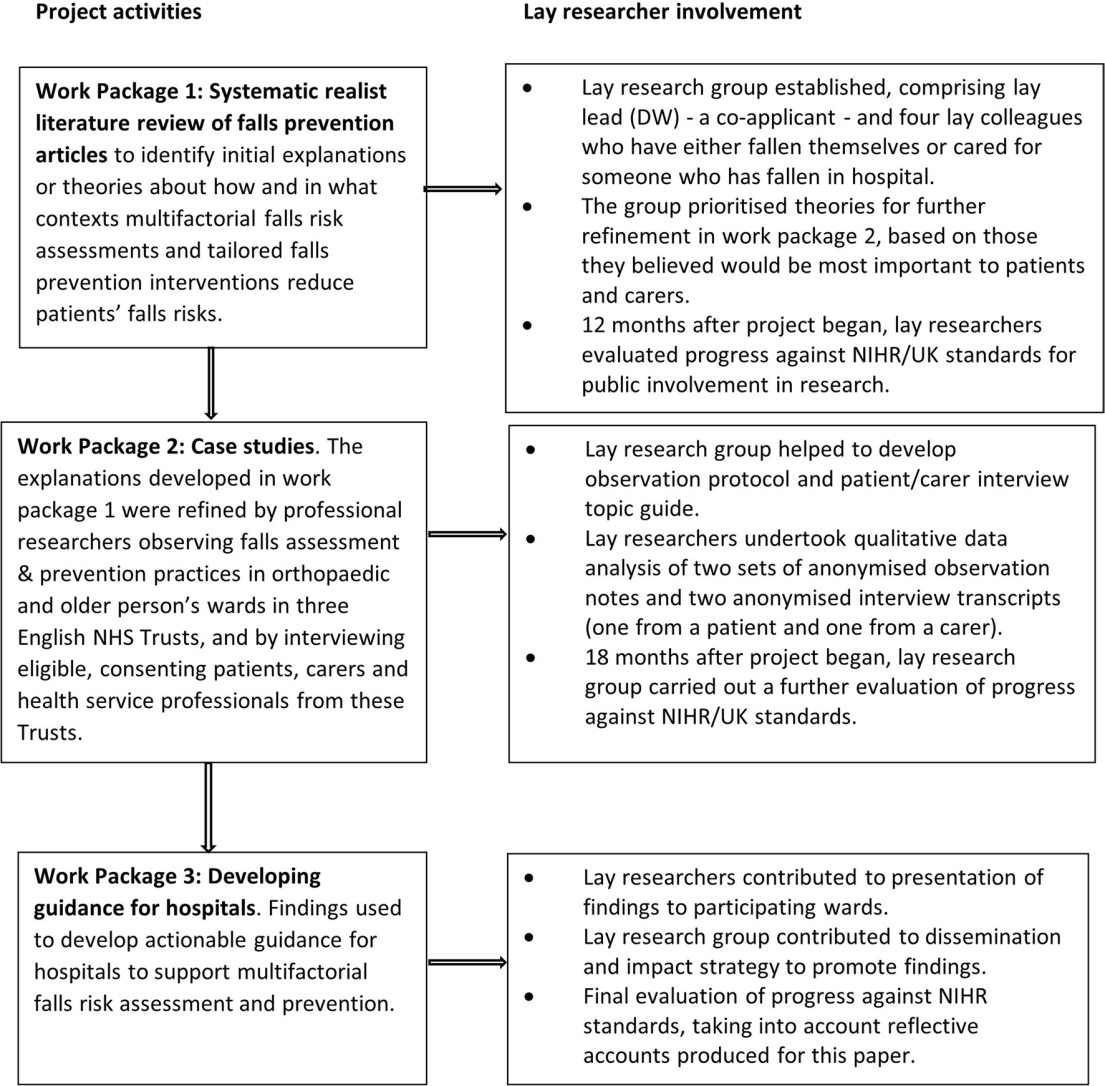
Diagram of project activities and lay researcher involvement

Briefly, the project used realist methods [16, 17] to explore what supports and constrains the implementation of multifactorial falls risk assessment and tailored falls prevention interventions in acute hospitals, in accordance with guidance from the National Institute of Health and Care Excellence [18], and examined how, why, in what contexts, and for whom these falls prevention interventions led to a reduction in patients’ falls risks. It was carried out in three work packages, with lay researcher input embedded at each stage. This involvement is summarised in Fig. 1 and comprised: in work package one, helping to focus the study on areas lay researchers believed would be most important to patients and carers; in work package two, developing data collection tools and undertaking qualitative data analysis of two sets of anonymised observation notes and interview transcripts (lay researchers did not themselves recruit participants or collect data and did not have access to study data other than to the notes and transcripts mentioned above); and contributing to the dissemination of findings in work package three. The final activity is still ongoing at the time of writing this paper, with further joint dissemination activities planned. In addition, in all three work packages the lay researchers evaluated how the project was taking patient and public perspectives into account, as described in the next section.

### Method for evaluating how the project took patient and public perspectives into account

The study protocol stated that progress in taking patient and public perspectives into account would be evaluated at six-monthly intervals by professional researchers working on the project and by a team of lay researchers recruited to work with the study, known as the lay research group. The group was headed by the lay lead (DW), a co-applicant of the project, and included four lay colleagues from diverse backgrounds (e.g. different ages, ethnicities and sex) who had either fallen themselves or who had cared for someone who had fallen in hospital (BI, ED, TF and JA). A professional researcher, LM, supported the group by setting up meetings, circulating papers and taking notes, as well as by offering advice and support throughout the project. For example, she advised on technical issues such as how to use software, and also sent informal emails to lay colleagues, updating them on wider progress with the project, checking on them if they were not well and so on. The project took place from 2020 to 2022, during the COVID-19 pandemic, and all lay research group meetings were held online, using the Microsoft Teams™ video-conferencing platform.

**Fig. 2:**
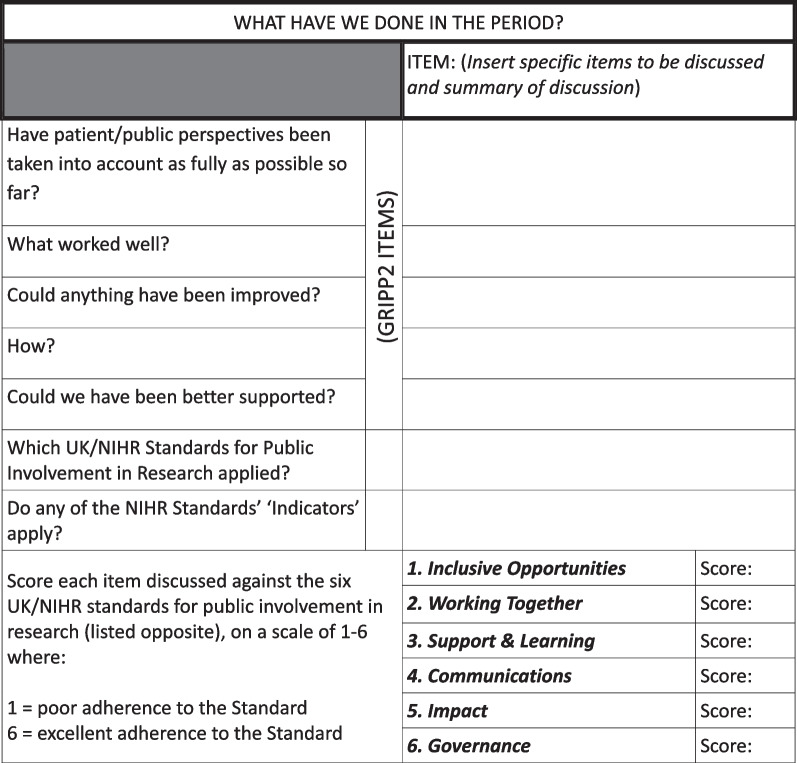
Evaluation sheet

The protocol stated that the evaluations of the extent to which the project took patient and public perspectives into account would be based on national standards for public involvement in research [7, 8]; given the expectation in those standards of working together, its precise form was developed by the lay researchers, once the project started. Three evaluations took place: the first was in May/June 2021; the second in February 2022, and the final, end-of-project evaluation was in October 2022. The evaluation method incorporated reflective questions derived from the Guidance for Reporting Involvement of Patients and the Public (GRIPP2) short form reporting checklist [19]. The questions were applied to discussion items, which were ‘scored’ by lay researchers when they met together to carry out the evaluations. Scores were allocated on a scale of 1–6 against whichever of the six national standards were deemed relevant [7, 8]. The standards are: (1) inclusive opportunities; (2) working together; (3) support and learning; (4) communications; (5) impact; and (6) governance. The scoring system was inspired by an evaluation model developed by the NIHR Yorkshire & Humber Patient Safety Translational Research Centre, with a score of one reflecting poor adherence to the standard and a score of six reflecting excellent adherence. An evaluation sheet was developed to capture these discussions: see Fig. 2. It included a section about whether any of the NIHR standard indicators applied to an item. These indicators were included in the 2018 NIHR version of the standards (7); in the 2019 UK-wide version of the standards (8), the indicators were replaced by questions users can ask themselves, to reflect on progress.

For the first evaluation in the summer of 2021, a comprehensive review was carried out. First, the lay research group met to evaluate progress and allocate scores (LM attended only to record results on the evaluation sheets and did not contribute to discussions), then the professional researchers (RR, NA and LM) met separately to carry out their own review (which LM again documented on evaluation sheets). Both groups decided on topics for discussion independently. Finally, the lay and professional researchers met together to review progress overall. In documenting this joint meeting, LM produced a summary of progress against each standard, which outlined the discussion and recorded the final scores, agreed in the joint meeting.

For the second and third evaluations in February and October 2022 respectively, a ‘lighter touch’ was used, in which the lay research group met to discuss whether anything had changed since the previous evaluation, and a summary sheet was produced. Group members considered that this more streamlined approach was appropriate at these later stages, because they had carried out the detailed groundwork in the first evaluation, and therefore had a good understanding of the project. Outcomes from all the evaluations are included in the results section, below, with an emphasis on outcomes relating to standard two of the national standards for public involvement in research, ‘working together’.

### Method for reflecting about working together

Both the process of producing this paper and the final evaluation of the project involved reflecting on working together. Before starting to write the paper, a meeting of the lay researchers and LM was held to agree its focus and how we would write it. We discussed models such as one author writing the whole paper and co-authors commenting on it or writing the paper collaboratively in a workshop. A middle way was chosen, with LM drafting the paper and others commenting, whilst also incorporating reflective statements written by co-authors. Both lay and professional researcher contributors were free to produce these reflective statements, focusing on what it felt like to work together as partners on the FRAMES project; what impact this had on each person and/or on the team or the project; and what they felt helped us to work together. Such flexibility about the nature and level of each person’s involvement (i.e. deciding whether or not to provide a reflective statement) was a key principle throughout the project, in keeping with the emphasis on inclusive opportunities in the UK standards [8]. Four of the five lay researchers decided to write a statement, as did a professional researcher. Examples from the statements are given in ‘boxes’ in the results section below, and they were also reviewed as evidence for the third and final evaluation of the extent to which the project took patient and public perspectives into account, in October 2022.

## Results

Working together in ways that value all contributions and that build and sustain mutually respectful and productive relationships is the second of the six UK standards for public involvement in research [7, 8]. As noted above, the most recent version [8] includes a series of questions within each standard, to help users reflect on the extent to which they meet it. In the following section, the questions for the second standard are used to structure findings about the processes that helped this team to work together, drawn from both the formal evaluations of how the project took patient and public perspectives into account, and from the reflective statements produced by lay and professional researchers, described above.

Has the purpose of public involvement been jointly defined and recorded and is there is a shared understanding of roles, responsibilities and expectations of public involvement?

**Table:** 

Box 1: A lay researcher reflects on not just ‘ticking a box’	
When I started working with the lay research group, I was concerned that the role might just be to comment on a few forms before and after submitting them to ethics, so the group would only help the researchers in ticking a box for their ethics application. I was affected by the falls that my father had and wanted to have an impact on preventing falls in the future. I was not sure, however, if the researchers would take our PPI views as carers or public contributors with good consideration. Luckily, this was the case	

The lay lead, DW, was involved in the initial development of the project. He advised on the patient and public involvement sections of the project bid, which was written by the principal investigator (RR) and then checked and edited by him. This set the tone for the project’s approach towards lay research; indeed, in the first evaluation RR commented that DW’s approach had ‘*completely transformed*’ her way of thinking about lay involvement in projects and had inspired her to apply this thinking to other projects (in several funding bids she has developed since the FRAMES project began, she has, for example, included plans for a lay research group involved in research processes such as data analysis, rather than a more conventional lay advisory group). DW also advised on an information sheet which was sent to lay people interested in joining, which explained the project’s aims and objectives, outlined lay researcher duties and included a brief post specification. Later, once lay researchers (BI, ED, JA and TF) were recruited, DW and LM provided an introductory PowerPoint presentation structured around: (1) people and groups/committees involved in the project, with photographs of individuals so lay colleagues knew what they looked like (especially important given the project began during a Covid lockdown when members were unable to meet in person); (2) an introduction to realist research methods used on the project^2^; (3) project and lay researcher tasks and timelines; and (4) next steps. Under the final item lay researchers were asked if they felt clear about how they were involved and what they expected to happen; what kind of training they thought would be helpful; and how they would like to receive it. They were invited to ask questions and make suggestions.

In their first evaluation, the lay researchers noted that this process had been fair and transparent, commenting: ‘*the role was clearly set out and explained*’ and adding: ‘*It feels ok to ask questions if you’re not sure about something and this has helped us all to contribute strongly, even in these strange pandemic times*’. Also at this stage in the project, DW asked project members (both lay and professional researchers, including all members of the project management group) to share informal ‘mini-CVs’. These short documents, often illustrated with informal snapshots, included a brief description of the person’s background relevant to the project; professional and/or lay experience; and, significantly, other interests. That section, in particular, was a powerful reminder that everyone on the project, whatever their background, was a person with hobbies, families, pets and so on. The professional researchers, in their evaluation, commented that the mini-CVs had: ‘*contributed to the inclusive culture of the project, and the feeling of being one team, with members bringing different skills and experiences: not ‘us and them’*’.

In the final evaluation in October 2022, thinking about their experiences during the project as a whole, the lay researchers discussed whether even more could be done during recruitment and induction to clarify the potential impact on lay people of being deeply involved in research that had profound significance for them. Although lay researchers knew they could talk about anything that was on their mind in the meetings or, separately, with DW or LM, they were not signposted to more formal sources of support (such as counselling services) and they felt such signposting could be a helpful part of induction in similar projects (see also recommendations in the **Conclusions** section below).

### Have the practical requirements and arrangements for working together been addressed?

Practical arrangements for working together were discussed from the beginning of the project in the introductory meeting and PowerPoint presentation, where, for example, lay researchers were asked how they preferred to be communicated with and how they liked to receive documentation. As a result, documents for meetings were sent both as pdfs and in Microsoft Word™, because not all members had access to the latter. Later, when the group engaged in data analysis, hard copies of anonymised documents were sent to their homes, because some members preferred to work on paper, given the volume of paperwork involved in that task.

The fact that meetings were held online using Microsoft Teams™ presented both practical problems and opportunities. At first some members had difficulty connecting to meetings. Having discussed this, it became clear that sending Teams invitations to diaries did not work well for people who did not use the Microsoft Outlook™ email program. Links to meetings were lost and people could not log in without them. In response, LM embedded meeting links in agendas for each meeting, in emails sent about a week before each meeting and again on the morning of the meetings. In their second evaluation, lay researchers commented that this simple adaptation avoided the first ten minutes of meetings being taken up with problems logging in, which they experienced in some other online meetings they attended.

Lay researchers thought they had managed to work well together online, without some of the social cues that face-to-face meetings can provide, because they treated each other with respect. They commented that the remote nature of the meetings supported this inclusive process, because it encouraged them to listen carefully and to give each other time to talk. Such meetings also removed the barrier of travel, which supported people who were carers or who were unwell (attendance was excellent: in eight meetings over two years, only two apologies were given, despite some members caring for others or having health concerns during this time), see also **Box 2**. As a result, having at first intended to meet in person once Covid restrictions ended, the group continued to meet online for the duration of the project.

**Table:** 

Box 2: A lay researcher reflects on the impact of remote meetings during the pandemic	
Extraordinarily all our meetings have been online due to Covid. This has meant that we haven’t had to travel, which many of us would have found difficult, or dress up or whatever, and we have had time to prepare. Any worries we may have had have been dealt with in advance. There has been a great deal of support and care. One help for me has been the ability to see faces and hear properly, as at times I need to lip-read and feel at a disadvantage in a large group with external noise	

### Have individuals’ influence, ideas and contributions been recognised and addressed?

In the evaluations, lay and professional researchers agreed that progress against this standard was strong, because of the inclusive, non-hierarchical teamwork approach established by the lay research group chairperson, DW, and the principal investigator, RR, who made it clear that individuals’ contributions were valued. For example, the very first meeting of the lay research group began by DW asking fellow lay researchers ‘Why did YOU say yes to this project?’ and then each member told their story (see **Box 3**).

**Table:** 

Box 3: A lay researcher reflects on ‘an atmosphere of caring and respect’	
Each of us had a different story to tell—all linked by a common thread. Recalling our varied experiences wasn’t easy, particularly in view of the lockdown situation we were in. But, this has been one of the best PPI experiences I have had. We genuinely worked as a team. Time was provided to share experiences and through these provide our observations on how best to approach the project. We were able to talk openly and honestly in an atmosphere of caring and respect	

Later, lay researchers were asked if they wished to share their stories more widely by providing blogs (https://www.bradford.ac.uk/health/research/frames/blog/). Acknowledging the personal impact of falls and the loss, grief and pain that may be associated with them, it was emphasised that such sharing was entirely up to each individual. One person, for example, having consulted with family members, decided not to provide a blog, whilst another started to write about their relative’s traumatic fall in hospital, but found it too distressing and instead wrote passionately about the importance of improving falls prevention practices. Flexibility was also offered in terms of blog production, with LM offering to ‘ghost-write’ lay researchers’ stories as blogs for those who did not have time or wish to write themselves (one lay researcher asked her to do this, while others preferred to write their own blogs). The blogs generated impact for the project, leading directly, for instance, to an invitation for the lay research group to give a presentation about falls prevention and their involvement in the study to a commissioning support group. Their impact was felt within the project too. As the professional researchers pointed out in their evaluation meeting, the blogs both expressed and reinforced a culture of mutual acceptance.

Another important way in which lay researchers’ ideas and contributions were embedded in the project was through their involvement in prioritising the study’s focus at the end of the first work package. The realist literature review undertaken at that stage of the project identified several explanations about what supports and constrains falls prevention practices, and both the lay research group and study steering committee were involved in deciding which of these would be developed in the next stage. The lay researchers rated the theories independently, rating most highly those they thought would be most important for patients and carers, before meeting together to discuss them in detail and producing a final, ranked list. The study steering committee also discussed and ranked the theories but gave the lay research group’s ideas precedence in determining the project’s next steps.

As impactful as this exercise was, it was also challenging for lay and professional researchers alike, requiring much reading and thought quite early in the project. One lay person reflected that she found it daunting and even wondered if she was capable of making a contribution, but she was encouraged to go on by the lay lead: ‘*He was so supportive and encouraging that I pushed myself to carry on. I trusted in his help and support*’ (Lay researcher reflective statement). As a result, she contributed strongly until the end of the project.

In the second evaluation in February 2022, lay researchers considered that progress had continued in this area, citing examples such as their involvement in developing data collection tools for the second work package, in particular a schedule for ethnographic observations of falls assessment and prevention practices on hospital wards, and topic guides for patient and carer interviews. Their feedback ensured that patient and public perspectives were at the heart of data collection, and it also had a more personal impact. A professional researcher, for example, explained in her reflective account that the lay feedback helped her to feel more confident as an ethnographer (see **Box 4**).

**Table:** 

Box 4: A professional researcher reflects on how working together helps her to be a better ethnographer	
As an ethnographer observing practice on busy NHS wards, I sometimes feel I am intruding. This discomfort (which comes from within me, and which I feel despite staff and patients being welcoming) can inhibit me as a researcher, because I think I do my best work when I have an open attitude and when I’m not expending energy on feeling like I’m in the way. Because I’d been with the lay researchers while they discussed the observation schedule, I felt almost as though they were with me when I was on the wards, advising me to take notice of this or think carefully about how I asked that. I felt less isolated as a result, and therefore more confident to observe non-defensively	

Lay researchers were also involved in analysing data. One person wrote movingly in a reflective statement about how this had given a feeling of connection to her father, who had fallen in hospital before he passed away, and a sense that she was helping to make it less likely that other patients would have the same experience (see **Box 5**).

**Table:** 

Box 5: A lay researcher reflects on the personal significance of involvement	
When the professional researcher anonymously shared with us some of the interviews, I felt like I was there at those sites, observing alongside her. I also had this strange feeling like I was making my father happy by contributing to this project, even after we lost him. I felt like he was there, sat at the corner of the room with a content smile on his face looking at me and the professional researcher. It felt like him saying: ‘Thanks for doing this, thanks for preventing others having falls at the hospital the way I did’. It felt like helping him even after he’s gone	

For others, however, data analysis was more challenging. One lay researcher, who had fallen in the past at home, resulting in a serious head injury, and who had come close to falling in hospital, was disturbed by the data. Having involved herself in the project to help change things for the better, the interview transcripts and ethnographic notes, with their vivid descriptions of day-to-day life on busy wards during the pandemic, gave her a sense of futility about the enormity of the challenges faced by the health service. As a result, she withdrew from some of her public contribution activities for a time (see **Box 6**).

**Table:** 

Box 6: A lay researcher reflects on challenges of coproduction	
When I received the first actual observation it had a real impact on me. I found it upsetting that situations in hospital settings had not really improved over the years; in fact, they seemed to be worse, probably due to Covid and the lack of carers on the wards [in the interests of infection control, hospital visiting was restricted during the period when many of the observations for the FRAMES study took place], despite the tremendous efforts of so many caring and concerned people. I am afraid this made me withdraw from many of the activities I had become involved in as I felt my family and social life was being affected by my concerns and I felt I could not do anything of value. Fortunately, after several weeks of respite some energy is returning and I am beginning to think about other ways that I may be able to help	

Colleagues on the project kept in touch and asked her how they could help, and they talked things through together. She was not offered more formal support, however, such as counselling. In their final evaluation in October 2022, the lay research group discussed the kind of support that lay people may need during projects if they feel distressed and agreed that contact details should be provided for support organisations like the Samaritans. They also reflected that it had been helpful to discuss feelings in the group and to prepare the reflective statements, which had enabled them to share and process their feelings. The lay researcher who had felt impacted by the data said support from group members had helped her, as had her decision to withdraw from public activities for a while as part of her self-care, and she welcomed the flexibility provided by the group to do so. In producing this paper and reflecting again on her experience, she confirmed that whilst she did not need counselling, owing to her strong inner resources and support from friends, family and colleagues on the project, it would be good practice to signpost people who are emotionally impacted by research to counselling, in case they do not have such sources of support.

In the final evaluation, the lay researchers also reflected on how the project had recognised their contributions overall. A point that emerged strongly was the impact of being recognised and accepted as individuals, which they felt had freed them to offer what they could. They felt valued and able to be themselves; one person, for example, talked about how their confidence had grown in other public endeavours. From this foundation of respect, they were able to enhance the project by, for example, attending the final presentation of results to the participating NHS Trusts and contributing to the discussion of impacts with researchers, clinicians and managers. Other ways in which they strengthened the project included their work to focus the study on areas that matter to patients and carers and the changes they made to data collection tools, which helped professional researchers ask meaningful questions in patient and carer interviews and look out for things on wards they might otherwise have missed, such as the role of non-clinical staff in falls prevention. Their contribution to data analysis enabled a more nuanced interpretation of study data, concerning, for example, the importance of staff getting to know patients when tailoring falls prevention activities to their individual needs.

### Have all the potential different ways of working together been explored, and have these plans and activities been developed together?

As noted above, different practical ways of working together were explored, such as LM ‘ghost-writing’ lay blogs when asked. However, more wide-ranging discussions also took place in the joint lay/professional researcher evaluation. They discussed whether lay input at the project development stage could be even more extensive, and agreed that, in future projects, one option could be to form an initial lay research group before the project was funded (knowing that funding might not be forthcoming), which would have time to discuss proposals in depth and ensure patient and public perspectives were embedded. Although limited one-off funding could sometimes be found for such work, lay researchers considered that even if no expenses were available, this would not necessarily be a barrier to involvement, provided the situation was explained transparently. Such involvement would improve the public relevance of a project, and thereby its chances of being funded, and reflects approaches to involving members of the public in the early stages of the research cycle recommended by the NIHR [20].

Finally, in terms of lay involvement in project management, DW was a member of the project management group and reported on matters of public involvement at each meeting. Notes of the meetings of the project management group and the study steering committee were copied to lay research group members. Towards the end of the project, in September 2022, a joint lay research group/project management group meeting was held to consider the final outputs of the study and its dissemination, providing an opportunity for both lay and professional researchers to discuss these and together agree on further work.

## Discussion

Drawing on lay and professional researcher evaluations of the extent to which this NIHR-funded applied health project took patient and public perspectives into account, and reflective accounts about what it was like for us to collaborate, we have shown how professional researchers and members of the public can work together, as envisaged in the public involvement and coproduction literature. We have provided evidence about how this was done in the FRAMES project by defining roles, responsibilities and expectations of involvement; addressing practical requirements and arrangements; recognising, using and celebrating individuals’ ideas and contributions; and exploring and jointly developing different ways to work together.

As we discovered, deep participation of this sort can be both immensely rewarding and challenging for research projects and individuals. In terms of positive impacts, the role of lay researchers in, for example, shaping data collection tools and analysing data arising from the application of those tools enriched the FRAMES project by identifying key areas for observation on hospital wards and helping to ensure professional researchers asked meaningful questions in patient and carer interviews. Positive impact was also felt at a personal level, both by a professional researcher, who reflected on how it helped her to carry out ethnographic observations less defensively, and by lay researchers, one of whom wrote about such involvement feeling like helping her late father, who had fallen twice in hospital. Other authors have pointed to profound but implicit impacts of this kind: Russell, Greenhalgh & Taylor [12], for example, report that by their very presence lay contributors ‘*may change the course of a discussion or attitudes to an issue in significant but subtle and hard to measure ways*’ (p.19). Noting challenges in quantifying impacts of this kind, they caution against simplistic or formulaic approaches to measuring such effects and discuss new ways of reporting that ‘*embrace the characteristics of complexity, for example, unpredictability, multiple interacting influences, inherent and dynamic tensions*’ (p.21). We think the evaluative and reflective approach developed by lay researchers for the FRAMES project does this, and we hope it provides a useful model for others.

Turning to challenges, the tensions and complexities inherent within collaborative work, mentioned by the above authors, were also experienced by FRAMES lay researchers. One person, for example, was upset by analysing vivid ethnographic accounts and interview transcripts and reflected on how, for a time, this caused her to step back from some of her work as a public contributor, because she thought she ‘*could not do anything of value*’ (Lay researcher reflective account). As Maguire and Britten [13] note, the expertise that lay people bring to research often draws on ‘*raw, painful and intimate experiences*’ (p.464). Whilst this can contribute strongly to the profundity, authenticity and validity of their contributions, it may also mean lay researchers can be powerfully impacted by research, and may even be retraumatised by it [21, 22]. As a team of co-authors and co-researchers, we have discussed this issue and concluded that narrative data of the kind found in qualitative research may be particularly likely to trigger emotions in this way, because of their first-person immediacy. Bearing this in mind, we endorse recommendations for professional and lay researchers to discuss openly the potential for emotional triggering before and during projects, encouraging each other to reflect on how they might be affected, secure in the knowledge that they are welcome to opt in or out at any time [13, 21]. We also commend the use of reflective statements among lay and professional researchers as a means of processing feelings, as we have done in the development of this paper and the final evaluation, because they helped us to identify, share and discuss personal responses that might otherwise have remained unsaid. Furthermore, in their final evaluation, the lay research group recommended that, in future projects, contact details for counselling and support organisations should be provided to lay contributors.

However, the lay researcher who was impacted negatively by the data on the FRAMES project responded not from a place of re-traumatisation, but rather from a sense of social responsibility (the very sense that had encouraged her to contribute to the project initially); she felt overwhelmed and even angered by the enormity of the pressures on hospitals and their staff and patients, described so vividly in the data. Whilst research team members are familiar with discussing the changes they hope projects will generate, less is said about the need to discuss the limits of change and the effects this can have on members. We think, however, that this aspect is as important as the potential for re-traumatisation and requires as much discussion and care, not least because altruism and a desire for social change or justice are common motivations for involvement in research for both lay and academic contributors.

Such discussions require supportive relationships, and these were instrumental on the FRAMES project in ensuring that lay and professional researchers alike were able to work productively, innovatively and safely together. This finding is echoed by several other authors, who write about developing safe spaces within which people can express themselves and collaborate to produce new knowledge [2, 22]. Based on evidence from the evaluations and reflective statements, we believe the FRAMES safe space was generated by the honest, respectful interaction of each of the people involved, not least the principal investigator, RR, and lay lead, DW, in their inclusive attitude to leadership and power-sharing and the lay researchers BI, ED, JA and TF in courageously using painful experience to generate insight and change.

The complementary roles of DW, as lay research group chairperson, and LM, as the professional researcher tasked to support the group, were also important, drawing, in DW’s case, on years of work in both healthcare and public involvement, and in LM’s case on methodological understanding, as well as previous experience as a counsellor. Although some other lay and professional researchers may not have such backgrounds, for us, the important point is the way they worked together to promote patient and public involvement in the research; for instance, LM documenting rather than attempting to shape or control the lay evaluations and DW advocating for lay perspectives at formal project committee meetings. Both individuals offered emotional support to lay colleagues and to each other. In this way, they jointly acted as process facilitators, a role recommended for effective patient partnership in health research agenda-setting [9]. Process facilitators support partnership using interpersonal skills and knowledge of group dynamic processes, and by teaching or explaining different perspectives to stakeholders. Ideally, they have no personal stake in the outcome, just as DW, although a lay person, has no personal experience of falling or caring for someone who has fallen, unlike the other lay researchers. It may be difficult for one person, whether a lay or professional researcher, to embody all these skills and qualities (LM, for example, may have had a role in facilitating the lay research group’s work, but as a researcher employed full-time on the FRAMES project, she was not independent). As a result, sharing responsibility for process facilitation, as DW and LM did, may be a useful model, provided that the sharers work closely and consistently together, whilst acknowledging each other’s specific roles.

### Strengths and weaknesses

A strength of the methods for working together outlined in this paper, and for evaluating and reflecting on these methods, is that they are examples of power-sharing [6], developed and implemented by lay and professional researchers in partnership. Caring, respectful relationships between these people made this possible, and we regard these as a further, fundamental strength of our approach; they are the ground on which everything else was built. However, we have talked as co-authors about whether these relationships may also have limited the evaluations in some ways, making both lay and professional researchers more inclined to give positive feedback and less inclined to be critical of people and a project to which they were emotionally attached. We have tried to counter that dynamic by reflexively considering and discussing our own potential for bias, but it may be that evaluations led by a truly independent process facilitator, of the kind recommended by Abma and Broerse [9], for example, could have achieved more objective results. We believe, however, that the potential benefits would likely be off-set by the reduced psychological safety felt by contributors towards a facilitator they did not know, and conclude that the inclusive and subjective approach used here has more to offer, provided it is implemented and reported as reflexively and transparently as possible.

We acknowledge, further, that more rigorous and streamlined processes could be developed. We could, for example, have drawn more extensively on formal methods to inform our approaches to evaluation and reflection, such as Gibbs’ reflective cycle [23]. The first iteration of the evaluative method, with separate meetings of lay and professional researchers, followed by a joint meeting, all of which were documented in detail, was comprehensive and valuable. It was also resource-intensive, and the following evaluations took a simpler form, whereby the lay research group alone reviewed changes since the last event. Other researchers may be able to develop even more robust and streamlined processes, and we hope our example may prove useful to them in this.

## Conclusions

In this paper we have shown how lay and professional researchers can work together to produce better research. To inform other lay and professional researchers, we provide recommendations about how to do this, based on our experiences (see Table 1). We have also shared an innovative approach to evaluating the extent to which projects take patient and public perspectives into account that others can use, with the potential for widespread impact.

**Table 1:** Recommendations for working strongly together

Developing research proposals	Consider involving a group of interested lay people when developing research proposals to enhance the public relevance of projects from the very beginning. As the NIHR advises [20], it should be made clear that funding processes can be lengthy and the research may not be funded, but seed-corn funding is sometimes available to pay lay expenses at this point
Being clear about lay contributors’ roles and the impact research can have on them	Provide a clear outline of lay contributors’ roles before projects begin and discuss these with people who are interested in the work, whilst allowing for lay contributors to develop their own ways of working. Although it may not always be possible to detail all aspects of the role upfront, it should at least be stated clearly that involvement in research can be emotionally challenging, especially in qualitative research, where lay colleagues may be exposed to vivid accounts that could be painful or overwhelming, perhaps during data collection or analysis
Supporting lay contributors	In recognition of the potential emotional challenges inherent in research involvement, formal sources of support (for example, counselling services) should be highlighted in lay role descriptions and the facilitators of lay groups should reiterate that these supports are available throughout the project. Informal support is also important, such as facilitators and group members asking how colleagues are feeling and offering to talk things through if they seem upset or withdrawn, sending supportive messages or a card, and keeping in touch regularly. On this project, we kept in touch with emails or telephone calls between meetings, especially if someone was experiencing challenges. The reflective statements, written by lay and professional researchers, were another, effective means of processing feelings because they encouraged us to think about our experiences and discuss them together. Finally, we recommend that professional researchers think carefully about the kind of data they expose lay colleagues to. Lay contributors’ wellbeing should come first, before any other objectives
Addressing power imbalances	Think about ways to address power imbalances between lay and professional researchers from the start. On the FRAMES project the mini-CVs were a simple but powerful way to establish an inclusive approach. Other mechanisms included sharing minutes of project management and steering group meetings with lay researchers and having a joint lay research group/project management group meeting. On a personal level, we tried to counter imbalances through warm, caring and respectful relationships between people
Addressing practical challenges	Find ways to address practical challenges: for example, in the FRAMES study, the simple technique of putting hyperlinks to Teams meeting in an email to lay researchers a few hours before online meetings avoided problems logging into meetings and made more time for collaborative work. Documentation can also be provided as pdfs and in Microsoft Word™, in case anyone does not have access to Word, and some people prefer to receive hard copies of documents
Facilitating lay groups	In our project, it was useful to have both the lay lead and a professional researcher supporting the lay researchers and acting as facilitators. To do this effectively, facilitators need to draw on relational and groupwork skills and knowledge of the research and its methods (in complementary ways: one person does not need to have all these attributes), and they must also have adequate time, as well as a mandate and a clear remit to do the work. Facilitators need to be sensitive to the feelings of group members, so that they are aware if anyone is struggling and needs further support
Building and celebrating relationships	Working strongly together is a relational undertaking and, accordingly, it is important to recognise and celebrate the importance of relationships from the start. For example, the first item on every FRAMES lay research group meeting was a social catch-up, and we gave this as much time and importance as any other agenda item because supporting strong relationships underpinned everything we did. Since relational undertakings are, by their nature, as diverse as the people who engage in them, each group should find ways that fit for them. Good relationships are also grounded on trust between group members. In this study we developed trust by keeping in touch regularly (both about work and social matters), being honest and open with each other and listening to each other

Finally, in recognition of his influence on the FRAMES study, and the way he fostered partnership throughout with clarity, kindness and insight, we leave the last word to the lay lead, DW. In his reflective statement, he talked about how members of the FRAMES project found ways of working that were right for them and likened the process to piecing a jigsaw together, as stated in **Box 7**. We think this is a fitting metaphor (in every sense of the phrase) for the way FRAMES lay and professional researchers worked together: like the pieces of a jigsaw members were connected and strengthened by their differences. In fact, it was because of those differences that they joined together so well, each making up an equally important part of the puzzle.

**Table:** 

Box 7: Working together is like constructing a jigsaw	
Each element of a jigsaw is unique in its qualities (colours, shape, position within the picture) but these qualities co-exist with those of every other piece. In the same way, the FRAMES research team members are all unique in their qualities but co-exist effectively. Perhaps they are an academic or clinician or lay person; perhaps their forte is reviewing literature or empirical methodology or participant observation; perhaps they knit in their spare time or climb mountains or play the piano. Some of these qualities really do apply to some of the FRAMES team and as the project progressed we found we worked more closely together *because* of our different interests, backgrounds and skill-sets, rather than being forced apart	

## Data Availability

Not applicable.

## Abbreviation

FRAMES: Falls prevention in older adults: using Realist Approaches to improve Multifactorial assEssment and interventionS
NIHR: National Institute for Health and Care Research

## Footnotes

1 A note about terminology: The title ‘lay researcher’ (rather than, for example, ‘service user or carer’ or ‘expert by experience’, to name a few alternatives) was proposed by the lay lead, DW, when the project was in development. In so doing, he wanted to address power imbalances between lay and professional researchers, using the term to indicate that lay people were active members of the research team, and not simply advisers or consultees. The lay researchers on this project agreed and like the term. However, we recognise that no label is ideal. The same is true of ‘professional researcher’, which we have used in this paper to distinguish research staff employed on the study from lay colleagues. Although not our intention, this term may also embody power imbalances (as may ‘academic researcher’). We acknowledge the difficulty of finding inclusive language that helps people work together as partners and encourage further debate and development,

2 Before the project began, another co-applicant of the project, NA, met with DW to provide background information about realist methods, to prepare him to work on a realist project.
